# Dosimetric effects of immobilization devices on SABR for lung cancer using VMAT technique

**DOI:** 10.1120/jacmp.v16i1.5217

**Published:** 2015-01-08

**Authors:** Jong In Park, Sung‐Joon Ye, Hak Jae Kim, Jong Min Park

**Affiliations:** ^1^ Program in biomedical Radiation Sciences Department of Transdisciplinary Studies, Seoul National University Graduate School of Convergence Science and Technology Seoul Korea; ^2^ Biomedical Research Institute Seoul National University Hospital Seoul Korea; ^3^ Department of Radiation Oncology Seoul National University Hospital Seoul Korea; ^4^ Department of Radiation Oncology Seoul National University College of Medicine Seoul Korea; ^5^ Institute of Radiation Medicine Seoul National University Medical Research Center Seoul Korea; ^6^ Center for Convergence Research on Robotics Advanced Institutes of Convergence Technology Suwon Korea

**Keywords:** immobilization device, stereotactic ablative radiotherapy, volumetric‐modulated arc therapy

## Abstract

The purpose of this study was to investigate the dosimetric effects of immobilization devices on the dose distributions of stereotactic ablative radiotherapy (SABR) for lung cancer using volumetric‐modulated arc therapy (VMAT) technique. A total of 30 patients who underwent SABR for lung cancer were selected retrospectively. Every patient was immobilized using Body Pro‐Lok with a vacuum bag customized for each patient body shape. Structure sets were generated to include the patient body inside the body structure with and without the immobilization device. Dose distributions, with and without the immobilization device, were calculated using identical VMAT plans for each patient. Correlations between the change in dose‐volumetric parameters and the MU fraction of photon beams penetrating through the immobilization device were analyzed with Pearson correlation coefficients (r). The maximum change in $D_{95\%},D_{100\%}$, and the minimum, maximum and mean dose to the planning target volume (PTV) due to the immobilization device were 5%, 7%, 4%, 5%, and 5%, respectively. The maximum changes in the maximum dose to the spinal cord, esophagus, heart, and trachea were 1.3 Gy, 0.9 Gy, 1 Gy, and 1.7 Gy, respectively. Strong correlations were observed between the changes in ${\text{PTV D}}_{95\%}$, the minimum, the maximum, and the mean dose to the PTV, the maximum dose to the esophagus and heart, and the MU fractions, showing values of r higher than 0.7. The decrease in dose to the target volume was considerable for lung SABR using VMAT technique, especially when MU fraction was large.

PACS number: 87.55.‐x

## I. INTRODUCTION

Stereotactic ablative radiotherapy (SABR) is a specialized form of radiotherapy which delivers a high dose of radiation within a small number of fractions, generally fewer than or equal to 5 fractions.^(1)^ Therefore, incorrect delivery of a dose in a single fraction may harm a patient more severely than in the conventional radiation therapy (RT). In addition, target margins for SABR are usually not applied or applied minimally to avoid normal tissue complications due to the delivery of a high dose.^(2)^ For these reasons, accurate tumor localization is necessary for SABR.^1^, ^2^ Therefore, a variety of immobilization devices are used for SABR.^(2)^

One of the most promising applications of SABR is for the treatment of inoperable early‐stage lung cancer or oligometastatic lesions to the lung, showing a tumor control rate roughly two times higher than that of conventional RT.^(3)^ When performing lung SABR, since the lung motion due to respiration is considerable, an appropriate target margin is needed to cover the target volume with the prescription dose. To reduce normal tissue complications, various strategies have been used to minimize the target volume margins, such as gating, active breathing control (ABC), and abdominal compression.^(1)^ Abdominal compression is generally achieved with an immobilization device specialized for SABR.^(4)^

Although immobilization devices are located between the patient body and the radiation source during RT, sometimes the devices are ignored in practice as the immobilization devices are thin and generally made of radio‐transparent material, such as carbon. In addition, large field of view (FOV) CT images, which sacrifice CT resolution, may be required during CT scanning in order to include the entirety of the immobilization devices. Otherwise, the immobilization devices might not be fully included in the CT images. However, commercial treatment planning systems (TPS) calculate dose distributions only for the materials contained in the body structure.^5^, ^6^ Therefore, if the immobilization device is not included in the body structure, the dosimetric effect of the immobilization device is ignored. Various studies on the effect of immobilization devices, as well as couch structures on dose distributions, have been performed.^7^, ^8^, ^9^, ^10^, ^11^, ^12^, ^13^, ^14^, ^15^, ^16^ These studies have mostly focused on the dose perturbation by the immobilization device in the buildup regions affecting skin dose.^7^, ^10^, ^11^, ^12^, ^13^, ^14^ Several studies have investigated the dose perturbation by couch structures,^8^, ^10^, ^15^, ^16^ but no study has yet addressed the dosimetric effects of immobilization devices on the delivered doses to the target volume and to the organs at risk (OARs) for SABR using the volumetric‐modulated arc therapy (VMAT) technique. Although VMAT has gained popularity for SABR due to its speed and ability to reduce normal tissue complications by delivering beams from numerous gantry angles,^(17)^ the dosimetric effects of the immobilization device on VMAT for SABR is unclear. Recently, American Association of Physicists in Medicine (AAPM) task group 176 (TG‐176) recommended contouring the immobilization devices in order to consider their dosimetric effects.^(18)^ Following their recommendations, we investigated the effect of commercial immobilization devices for lung SABR on dose distributions delivered with VMAT technique. Assuming that the commercial dose calculation algorithm is sufficiently accurate,^19^, ^20^, ^21^ we compared the dose distribution calculated with the immobilization device to that calculated without it. The dose‐volumetric parameters were calculated and compared. The correlations between the changes in dose‐volumetric parameters due to the immobilization device and the fraction of monitor units (MU) of beams passing through the immobilization device were analyzed.

## II. MATERIALS AND METHODS

### A. Patient selection, simulation, and contouring

For this study, a total of 30 patients who underwent SABR for lung cancer in our institution were randomly selected retrospectively. All patients underwent four‐dimensional CT (4D CT) scans using a Real‐time Position Management System (RPM, Varian Medical Systems, Palo Alto, CA) and Brilliance CT Big Bore (Philips, Cleveland, OH). The slice thickness was 2 mm. During 4D CT scanning, all patients were immobilized using Body Pro‐Lok system (CIVCO, Orange City, IA) with a vacuum bag customized for each patient body shape (EZ‐FIX, Arlico Medical, Seoul, Republic of Korea). The 4D CT images were reconstructed using the phase‐binning algorithm (Philips, Cleveland, OH) and imported into the Eclipse system (Varian Medical Systems, Palo Alto, CA). Target volumes were contoured on each phase of the CT images, and they were aggregated on the CT images at 80% phase. The summation of the contoured target volumes was defined as the ITV, and the clinical target volume (CTV) was defined to be the same as the ITV. The planning target volumes (PTV) were defined with isotropic margins of 3, 5, and 7 mm from each CTV. The magnitude of the margin was determined by considering the proximity of the OARs to the PTV. The spinal cord, esophagus, heart, trachea, rib, and both lungs were contoured as OARs. As the Eclipse system calculates the dose distribution only for the region inside the body structure, we created three different structure sets for each patient to investigate the effects of the immobilization device on the dose distributions. The first structure set included only the patient body inside the body structure without the immobilization device. The second structure set included the patient body, the immobilization device including Body Pro‐Lok, and the vacuum bag inside the body structure. The third structure set included only the patient body and only Body Pro‐Lok. This structure set was generated to investigate the contribution of Body Pro Lok in attenuating the treatment beams without the vacuum bag.

### B. VMAT plans for lung SABR

**Figure 1:**
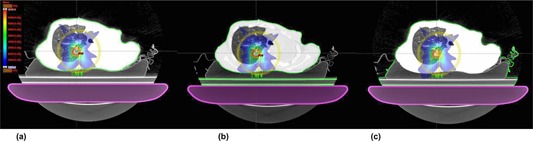
Body structures including patient body without the immobilization device (a), with the only Body Pro‐Lok (b), and with the Body Pro‐Lok as well as the vacuum bag (c) are shown. The body structures are indicated with green line.

All VMAT plans were generated on the CT images without the immobilization devices using the Eclipse system with 6 MV flattening filter‐free (FFF) beams. TrueBeam STx (Varian Medical Systems) with high definition multileaf collimators (HD‐MLC, Varian Medical Systems) were used for each VMAT plan. The prescription dose was 48 Gy (12 Gy/fraction) for 18 cases, 54 Gy (13.5 Gy/fraction) for 9 cases, and 60 Gy (15 Gy/fraction) for 3 cases. In order to reduce the dose to the contralateral lung, single or two partial arcs were used for the planning, according to the tumor locations.^(22)^ Coplanar arcs were used for all VMAT plans. Detailed information on all VMAT plans and tumor locations is summarized in Table 1. The numbers of VMAT plans with beams passing through only the vacuum bag, passing through the vacuum bag as well as the Body Pro‐Lok, and passing through no immobilization devices were 6, 21, and 3 cases, respectively. The couch structure provided by the manufacturer in the Eclipse system was always included during planning. The CT numbers of the couch structure were adjusted in order to match the measured values of beam attenuation to the calculations in the TPS. For optimization, the progressive resolution optimizer 3 (PRO3, ver.10, Varian Medical Systems) was used. For dose calculation the anisotropic analytic algorithm (AAA, ver.10. Varian Medical Systems) was used with a calculation grid of 1 mm. At least 95% of the prescription dose was delivered to 100% volume of the PTV except in one case. In this case, the tumor was located near the heart, and the $V_{95\%}$ was 44.2% to reduce the dose to the heart. Dose distributions were also calculated for the other two situations, with whole immobilization device and only with Body‐Pro‐Lok inside the body structure, using the same VMAT plan (Fig. 1).

**Table 1:** Summary of VMAT plan information

*Patient Number*	*Number of Arcs*	*Arc Type*	*Tumor Location*
1	1	Partial(>half)	Right upper
2	2	Partial (half)	Left upper
3	2	Partial(>half)	Right upper
4	2	Partial(>half)	Left upper
5	1	Full	Left lower
6	1	Full	Left lower
7	1	Full	Right lower
8	1	Full	Left lower
9	1	Partial(>half)	Left middle
10	1	Partial(>half)	Right upper
11	1	Partial (half)	Left middle
12	1	Partial (half)	Right middle
13	1	Partial (half)	Left lower
14	1	Partial (half)	Right lower
15	2	Partial (half)	Right lower
16	1	Partial (half)	Right middle
17	2	Partial (half)	Right middle
18	2	Partial(>half)	Left upper
19	2	Partial (half)	Right lower
20	1	Partial (half)	Right middle
21	2	Partial (half)	Left upper
22	2	Partial (half)	Right middle
23	1	Partial (half)	Right middle
24	2	Partial (half)	Right middle
25	2	Partial (half)	Right lower
26	2	Partial (half)	Right middle
27	2	Partial (half)	Left lower
28	2	Partial (half)	Left lower
29	2	Partial (half)	Left middle
30	1	Partial (half)	Right middle

Partial=partial arc,Full=full arc,half=half arc,>half=partial arc larger than half arc.

### C. Changes in dose‐volumetric parameters due to the immobilization device

For the PTVs, the volume of PTV receiving 95% and 100% prescription dose ($V_{95\%}$ and $V_{100\%}$), the dose delivered to at least 95% and 100% of the PTV ($D_{95\%}$ and $D_{100\%}$), the minimum dose, the maximum dose, and the mean dose to the PTV were calculated. For OARs, the maximum dose to the spinal cord, esophagus, heart, trachea, $V_{30\%}$ of the rib, and the mean dose to the lung were calculated. The calculated dose‐volumetric parameters from the structure set without the immobilization device were compared to those from the plans with the whole immobilization device and to those with only the Body Pro‐Lok. The statistical significances of the changes in dose‐volumetric parameters between the cases with the whole immobilization device and the case with only Body Pro‐Lok were analyzed using a paired *t*‐test.

### D. Correlation between the differences in dose‐volumetric parameters and the fractions of beams penetrating through the immobilization device

We calculated the attenuated MU fraction (%) as follows.

(1) $$attenuated\;MU\;fraction(\%)=\frac{\sum_{j=1}^{k}Arc_{j}\sum_{i=1}^{N_{cp}}MU_{i}(1-e^{-\mu x_{i}})}{Total\;MU}\times 100$$

where, μ is the linear attenuation coefficient of water at the mean energy of 6 MV FFF photon beam (0.0504 cm^‐1^), $x_{i}$ is the water‐equivalent distance of the immobilization device for the photon beam at the ith control point $(\text{CP}),{\text{MU}}_{i}$ is the MU at the ith CP, $N_{\text{cp}}$ is the number of CPs in an arc, ${\text{Arc}}_{j}$ is the jth arc, and *k* is the total number of arcs in a VMAT plan. As the degree of attenuation by the immobilization device increased, the attenuated MU fraction increased. If there are no photon beams passing through the immobilization devices, the value of attenuated MU fraction is 0.

We used Pearson correlation coefficients (r) to investigate the correlations between the differences in dose‐volumetric parameters due to the immobilization devices and the attenuated MU fractions. The statistical significance of correlations was analyzed and the corresponding p‐values calculated.

### E. Measurements of attenuation by the immobilization device

Attenuation was measured with a Farmer‐type ion chamber (type 30010; PTW, Freiburg, Germany) in a Solid Water phantom (Gammex, Middleton, WI) at a depth of 5 cm. The ion chamber was always located at the isocenter and the gantry angle was 180° in the international electrotechnical commission (IEC) scale. The field size was $10\times 10\;{\text{cm}}^{2}$ and 100 MU was delivered using a 6 MV FFF beam of TrueBeam STx. The first measurement was performed without the immobilization device, the second was performed including only the Body Pro‐Lok, and the third was performed including both vacuum bag and Body Pro‐Lok. The attenuation by the Body Pro‐Lok was calculated as follows:

(2) $$Attenuation_{BPL}(\%)=\frac{Meas_{couch}-Meas_{couch+BPL}}{Meas_{couch}}\times 100$$

where ${\text{Attenuation}}_{\text{BPL}}$ is the attenuation by the Body Pro‐Lok, ${\text{Meas}}_{\text{couch}}$ is the measured value with couch, and ${\text{Meas}}_{\text{couch}+\text{BPL}}$ is the measured value with both the couch and Body Pro‐Lok.

Attenuation by both Body Pro‐Lok and vacuum bag was calculated as follows:

(3) $$Attenuation_{ID}(\%)=\frac{Meas_{couch}-Meas_{couch+ID}}{Meas_{couch}}\times 100$$

where ${\text{Attenuation}}_{\text{ID}}$ is the attenuation by both Body Pro‐Lok and the vacuum bag, and *Meas_couch+ID_* is the measured value with the couch, Body Pro‐Lok, and the vacuum bag.

## III. RESULTS

### A. Changes in dose‐volumetric parameters due to the immobilization device

Table 2 summarizes the changes in dose‐volumetric parameters of VMAT plans for lung SABR due to the whole immobilization device and due to Body Pro‐Lok alone. For PTVs, the maximum, minimum, and average values of the differences in the ${\text{PTV V}}_{95\%},{\text{PTV V}}_{100\%},{\text{PTV D}}_{95\%},{\text{PTV D}}_{100\%}$, the minimum dose, the maximum dose, and the mean dose to the PTV are shown (n=30). The volume of PTVs in this study ranged from 4.2 cm^3^ to 61.5 cm^3^. For OARs, the maximum, minimum, and average values of the differences in the maximum dose to the spinal cord (n=30), esophagus (n=30), heart (n=27), and trachea (n=21); as well, the $V_{30\%}$ of the rib (n=27) and the mean dose to the lung (n=30) are also shown. All dose‐volumetric parameters decreased when the immobilization device was included in the calculation.

**Figure 2:**
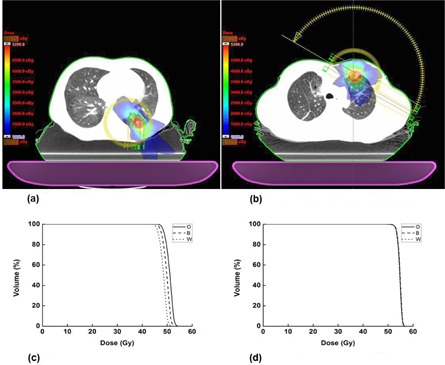
The case of the maximum changes in dose‐volumetric parameters for target volume due to immobilization devices (a) and corresponding changes in dose volume histograms (DVHs) of the target volumes due to immobilization device (c) are shown (O=only patient body,B= patient body with the Body Pro‐Lok, W= patient body with the Body Pro‐Lok as well as the vacuum bag). The case of no change in dose‐volumetric parameters for target volume (b) and their corresponding changes in DVHs of target volumes (d) are also shown.

For the target volume, the maximum difference due to the whole immobilization device was 8% for $V_{95\%}$. In the case of the maximum change in $V_{100\%}$, the value of the change was 83%. This value was exaggerated since it was expressed in percent value; in this case, the absolute value of the change was 0.73 cm^3^. The changes in $D_{95\%}$, and the maximum and mean dose to the PTV, ranged up to 5%. The change in $D_{100\%}$ ranged up to 7%. The values of $V_{95\%},V_{100\%},D_{95\%},D_{100\%}$, the minimum, maximum, and the mean dose to the PTV with Body Pro‐Lok alone were reduced by 2%, 52%, 2%, 6%, 4%, 4%, and 2%, respectively. The gantry rotation angles and dose‐volume histograms (DVHs) of the cases with the maximum and the minimum amount of changes are shown in Fig. 2. The attenuated MU fractions were 1.8% in the case of the maximum change, and 0% in the case of the minimum change. Since the attenuated MU fractions of each VMAT plan were varied, ranging from 0% to 1.8%, the values of the average differences were not large, showing less than 2.0% even with the whole immobilization device, except $V_{100\%}$ which had an exaggerated percent value.

**Table 2:** The changes in dose‐volumetric parameters due to immobilization devices

		*Maximum Diff*.		*Minimum Diff*.		*Average Diff*.		
	*N*	*B*	*W*	*B*	*W*	*B*	*W*	*p*
*Target Volume*								
$V_{95\%}\;(\%)$	30	2.5	8.3	0.0	0.0	0.03±0.75	1.71±1.71	0.042
$V_{100\%}\;(\%)$	30	51.8	82.7	0.3	0.7	4.69±12.4	22.1±22.1	<0.001
$D_{95\%}\;(\%)$	30	2.4	5.0	0.1	0.1	0.94±0.78	2.01±1.29	<0.001
$D_{100\%}\;(\%)$	30	6.2	7.3	0.0	0.0	1.06±1.45	1.90±1.78	0.000
Min. (%)	30	4.3	4.3	0.0	0.0	0.93±1.10	1.77±1.49	<0.001
Max. (%)	30	4.2	5.0	0.0	0.0	0.98±1.12	1.89±1.36	<0.001
Mean (%)	30	2.4	5.0	0.0	0.1	0.90±0.80	2.00±1.30	<0.001
*Organ at Risk (OAR)*								
Max. to spinal cord (cGy)	30	57.6	129.6	0.0	0.0	6.7±14.2	11.6±24.0	<0.001
Max. to esophagus (cGy)	30	52.8	91.2	0.0	0.0	11.6±16.3	20.9±26.4	<0.001
Max. to heart (cGy)	27	57.6	102.6	0.0	0.0	5.9±48.8	31.6±34.1	<0.001
Max. to trachea (cGy)	21	110.4	168.0	0.0	0.0	13.2±27.1	22.5±39.1	0.020
$V_{30\%}$ of rib (%)	27	13.0	27.5	0.0	0.0	2.2±5.4	6.6±12.4	<0.001
Mean to lung (cGy)	30	14.4	16.2	0.0	0.0	1.3±4.1	5.2±4.7	<0.001

N=number of cases; diff.= differences between structure set with and without immobilization devices; B=with Body Pro‐Lok alone; W=with Body Pro‐Lok and vacuum bag; $V_{n\%}=$ volume received at least n% prescription dose; $D_{n\%}=$ the minimum dose delivered to n% volume of the target volume; Min.=the minimum dose; Max.=the maximum dose; Mean=the mean dose.

For OARs, $V_{30\%}$ of the rib was decreased by up to 27.5% due to the whole immobilization device (60.5 cm^3^ in absolute value). The maximum differences in the maximum dose to the spinal cord, esophagus, heart, and trachea with the whole immobilization device were 1.3 Gy, 0.9 Gy, 1 Gy, and 1.7 Gy, respectively. These values with only the Body Pro‐Lok were reduced to 0.6 Gy, 0.5 Gy, 0.6 Gy, and 1.1 Gy, respectively. The dose‐volumetric parameters decreased, on average, by less than 0.32 Gy with the whole immobilization device, while the parameters with only Body Pro‐Lok were decreased by less than 0.14 Gy, on average.

Accounting for the whole immobilization device resulted in larger deviations from the original plan than accounting for the Body Pro‐Lok alone. The differences were caused by the attenuation in the vacuum bag. Since all p‐values for the differences between the changes with the whole immobilization device and Body Pro‐Lok alone were less than 0.02, the contribution of the vacuum bag to the attenuation of the treatment beam was also considerable.

### B. The correlation between the differences in dose‐volumetric parameters and the attenuated MU fractions

**Figure 3:**
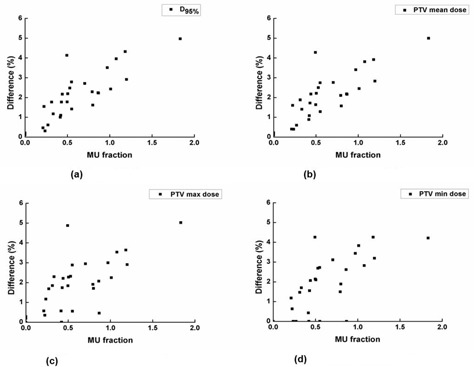
The attenuated MU fraction (%) vs. changes in $D_{95\%}$ of the planning target volume (PTV) (a), the mean dose to the PTV (b), maximum dose to the PTV (c), and minimum dose to the PTV (d) are shown.

**Figure 4:**
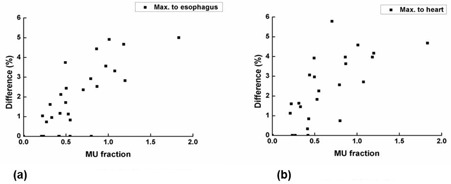
The attenuated MU fraction (%) vs. changes in the maximum dose to the esophagus (a) and heart (b) are shown.

The Pearson correlation coefficients (r) between the difference in dose‐volumetric parameters by the whole immobilization device and the attenuated MU fractions are shown in Table 3, along with corresponding p‐values. Strong correlations with statistical significances were observed in $D_{95\%}$ of PTV(r=0.842 and p<0.001), the minimum dose to the PTV(r=0.725 and p<0.001), the maximum dose to the PTV(r=0.701 and p<0.001), the mean dose to the PTV(r=0.824 and p<0.001), the maximum dose to the esophagus (r=0.785 and p<0.001), and the maximum dose to the heart (r=0.745 and p<0.001). Plots of the attenuated MU fraction vs. ${\text{PTV D}}_{95\%}$, the mean dose, maximum and minimum dose to the PTV are shown in Fig. 3. Plots of the attenuated MU fraction vs. the maximum dose to the esophagus and the heart are shown in Fig. 4. Considerable correlations with statistical significances were observed in $D_{100\%}$ of PTV(r=0.677 and p<0.001) and $V_{30\%}$ of rib (r=0.633 and p<0.001).

**Table 3:** Correlations between the dose‐volumetric differences due to whole immobilization devices and the MU fractions passing through those immobilization devices

	*N*	*r*	*p*
*Target Volume*			
V	30	0.067	0.725
V	30	0.308	0.098
D	30	0.842	<0.001
D	30	0.677	<0.001
Min.	30	0.725	<0.001
Max.	30	0.701	<0.001
Mean	30	0.824	<0.001
*Organ at Risk (OAR)*			
Max. to spinal cord	30	0.370	0.044
Max. to esophagus	30	0.785	<0.001
Max. to heart	27	0.745	<0.001
Max. to trachea	21	0.060	0.800
$V_{30\%}$ of rib	28	0.633	<0.001
Mean to lung	30	0.488	0.006

N=number of cases; r=Pearson correlation coefficient; $V_{n\%}=$ volume received at least n% prescription dose; $D_{n\%}=$ the minimum dose delivered to n% volume of the target volume; Min.=the minimum dose; Max.=the maximum dose; Mean=the mean dose.

### C. Measurements of attenuation by the immobilization device

The magnitude of attenuation by the whole immobilization device, including both Body Pro‐Lok and the vacuum bag, was 5.4%. The attenuation with the Body Pro‐Lok alone was 3.7%, showing that the major cause of attenuation was the Body Pro‐Lok.

## IV. DISCUSSION

The magnitude of changes in dose‐volumetric parameters increased as the result of increasing the attenuated MU fractions. Considerable correlations with statistical significance were observed between the attenuated MU fractions and the magnitudes of changes in dose‐volumetric parameters of the target volume, which suggests that the decrease in dose to the target volume was induced by the immobilization device. This could impair the tumor control rate, resulting in negative effects on treatment efficacy. The measurement results with the immobilization device decreased by an amount similar to that calculated by the TPS.

Since the introduction of VMAT to the field of radiation therapy, the effects of the couch structure on dose distributions have been investigated in several studies.^6^, ^23^, ^24^ Those studies have shown that the couch structure can induce considerable changes in the dose distributions of VMAT. Popple et al.^(25)^ showed that the couch structure could reduce dose to the isocenter by 5.8%. Vanetti et al.^(5)^ demonstrated that PTV coverage could be reduced by the couch structure by a clinically considerable level. Similarly, our results showed that the dose‐volumetric parameters of the target volume could be reduced by up to 5% due to the presence of the immobilization devices during VMAT for SABR. Although there were also changes in the parameters for OARs, since the changes in dose‐volumetric parameters in this study always decreased, the dosimetric effects of immobilization devices on OARs were not problematic.

Previous studies have shown that the skin dose from a 6 MV photon beam could be increased by up to 22% and 43% due to 2.5 cm and 10 cm thick vacuum bags, respectively.^13^, ^26^ However, it has been shown that skin dose is not critical in the case of VMAT for lung SABR because VMAT delivers beams from numerous directions, preventing a concentration of high dose in a specific region.^(27)^ In this study, skin dose enhancement was also observed to be noncritical (data are not shown). The more critical change caused by the immobilization device was the reduction in dose to the target volume. This study showed that the dose to the PTV could be reduced by up to 5% due to the immobilization devices for lung SABR. Since the use of both immobilization devices and the VMAT technique are highly recommended for lung SABR,^(2)^ the combination effect should be considered carefully.

To include the entirety of the immobilization device into the body structure, a large FOV may be needed, depending on the type of immobilization device. Such a large FOV can be achieved with a sacrifice in the resolution of CT images. Because the target volume of lung SABR is generally small (from 4.2 cm^3^ to 61.5 cm^3^ in this study), low resolution in CT images could potentially be a problem. A further problem is that low‐resolution CT images may also induce a change in volume of the fibrosis region during contouring.^(28)^ If the image resolution cannot be sacrificed, a careful approach is needed since the entirety of the immobilization device might not be included in the CT images.

The CT numbers of Body Pro‐Lok and the vacuum bag were in the range of ‐700 to 300 and ‐970 to ‐900 in this study, respectively. Gray et al.^(29)^ demonstrated the inaccuracy of the AAA algorithm when immobilization devices and large air gaps were present. In addition, recent studies have evaluated the performance of the AAA algorithm in terms of tissue inhomogeneity corrections for lung SABR.^30^, ^31^ Therefore, a careful approach is needed to consider the attenuation when including immobilization devices into the body structure. Although we used the AAA algorithm, which may be inaccurate for dose calculation in the situation of this study, the water‐equivalent thicknesses of the Body Pro‐Lok penetrated by photon beams were not considerable; therefore, the inaccuracy of AAA would not be as severe as in Gray et al.^(29)^ The degree of inaccuracy of the commercial dose calculation algorithm used in this study will be investigated as a future work. In this study we demonstrated the existence of considerable dosimetric differences due to immobilization devices. We confirmed that those dosimetric differences were due to the immobilization devices by correlation analysis with the attenuated MU fractions.

Although the commercial immobilization devices are thin and their CT numbers are small, they reduced the prescription dose to the target volume by a considerable amount. When using the VMAT technique, especially with posterior partial arcs, the immobilization devices should be included in the body structure for accurate dose calculation.

## V. CONCLUSIONS

We evaluated the dosimetric effects of widely clinically used commercial immobilization devices on the dose distributions of VMAT SABR for lung cancer. The reduction in dose to the target volume was considerable, especially when the MU fraction of beams passing through the immobilization devices was large. The decrease in the mean dose to the target volume could be up to 5% and was 2%, on average. The immobilization devices should be contoured and accounted for in dose calculations for good treatment efficacy.
